# Rhizosphere domestication enhances root colonization and plant growth promotion performance of *Bacillus velezensis* SQR9

**DOI:** 10.3389/fmicb.2025.1638130

**Published:** 2025-08-05

**Authors:** Zhengqi Wang, Yike Zhao, Jiahui Shao, Jingjing Wang, Weibing Xun, Xinli Sun, Zhihui Xu, Youzhi Miao, Guidong Huang, Derui Liu, Ruifu Zhang, Qirong Shen, Nan Zhang

**Affiliations:** ^1^Jiangsu Provincial Key Lab for Solid Organic Waste Utilization, Key Lab of Organic-Based Fertilizers of China, Jiangsu Collaborative Innovation Center for Solid Organic Wastes, Educational Ministry Engineering Center of Resource-Saving Fertilizers, Nanjing Agricultural University, Jiangsu Provincial Key Laboratory of Coastal Saline Soil Resources Utilization and Ecological Conservation, Nanjing, China; ^2^Department of Food Science, Foshan University, Foshan, China; ^3^Hubei Yishizhuang Agricultural Technology Co., Ltd., Yichang, China; ^4^Hubei Jiamachi Ecological Agriculture Co., Ltd., Yichang, China

**Keywords:** rhizosphere domestication, experimental evolution, PGPR, microbial fertilizers, sustainable agriculture

## Abstract

The overuse of chemical fertilizers has caused severe soil degradation and environmental pollution, necessitating sustainable alternatives such as microbial fertilizers containing plant growth-promoting rhizobacteria (PGPR). However, application of laboratory-developed microbial inoculants usually reveals impaired performance, attributing to complicated field conditions including plant genotype, soil property, and interaction with indigenous microbiota. Currently, traditional microbial breeding methods such as random mutagenesis and genetic engineering, could not be so appropriate for screening agents with comprehensive phenotypes (e.g., root colonization and plant growth-promotion effects). In the present study, we developed a rhizosphere domestication strategy for PGPR strain *Bacillus velezensis* SQR9, involving 20 cycles (approximately 160 generations in total) of *in situ* transfer and evolution in pepper rhizosphere. Evolved strains achieved 1.5 ~ 2.9-fold greater root colonization than the ancestral strain. A three-step phenotypic screening of 45 evolved strains firstly identified 29 candidates showing enhanced indole-3-acetic acid (IAA) production, biofilm formation, or siderophore production compared to the ancestor. Subsequent screening picked 6 strains with superior plant growth-promoting effects than the ancestor in hydroponic system. Final pot experiment confirmed the evolved strain 9P41 as the optimal performer, of which the inoculated pepper plants exhibiting 11.4% greater height, 28.7% longer roots, 21.0% higher aboveground biomass, and 29.1% increased underground biomass compared to plants treated with the ancestor. Genomic resequencing identified adaptive mutations in *mlnD*, *smc*, and *fhuC* genes are potentially associated with phenotypic improvements of strain 9P41. This rhizosphere adapted domestication strategy successfully breed evolved strains with improved plant growth-promoting efficacy, providing a novel solution for developing microbial inoculants and biofertilizers needed in sustainable agriculture.

## 1 Introduction

Despite the important contribution of chemical fertilizers to global food production, their long-term excessive application, especially in intensive agriculture areas, has caused critical issues. These include soil degradation, ecological disruption, and nitrogen-phosphorus pollution (Fitter, 2011). Under the global demand of agricultural sustainable development, microbial fertilizers are receiving attention for their dual capacity of reducing chemical inputs while restoring soil health (Elizabeth et al., 2021). These products usually employ plant growth-promoting rhizobacteria (PGPR) to improve nutrient cycling, produce phytohormones that enhance crop fitness, and inhibit plant pathogens (Ashok et al., 2024; Laura et al., 2019; Chandra et al., 2021; Nan et al., 2023). Unfortunately, the inconsistent performance of microbial fertilizers under complex field conditions has become a major bottleneck of this industry (Imran et al., 2019; Batstone et al., 2020; Vittorio and Cristina, 2021). For example, bacterial strains demonstrate significant growth-promoting and antimicrobial properties *in vitro*, yet their efficacy varies markedly in field conditions, which depends on host plant compatibility, fluctuating soil pH, temperature shifts, and competition with native microbial communities (Gurska et al., 2009; Jiakang et al., 2022). Therefore, it is of great demand to breed microbial strains that can effectively and consistently promote plant growth and suppress soil-borne diseases in complex agricultural environments.

Microbial breeding involves human-guided optimization of genetic traits to enhance their application potential in industrial, agricultural, and pharmaceutical areas. Common strategies mainly include random mutagenesis, genetic engineering, and directed domestication (Kang Lan and Tuck Seng, 2013; Mike et al., 2021; Neha and George, 2021). Random mutagenesis relies on physical (e.g., UV or X-rays), chemical (e.g., nitrosoguanidine, ethyl methanesulfonate), or biological (e.g., transposon insertion) methods to induce random mutations. Despite the effective application of mutagenesis in industrial microbe improvement, the randomness of mutation and high-cost of evaluating target phenotypes limits its efficiency in breeding microbial agents used in complex environment, such as excellent root-colonizer or plant growth-promoter that applied in field conditions (Ho Joung et al., 2020). Genetic engineering employs tools like CRISPR/Cas9 to introduce specific gene mutations, enabling precise modifications for pharmaceutical protein synthesis (Shen et al., 2020; Zhang et al., 2022; Reisenbauer et al., 2024). However, construction of these engineered agents requires a thorough understanding of the molecular mechanisms involved, also their release in agriculture still faces potential biosafety risks and legal restriction. Comparatively, directed domestication (also known as evolutionary experiment) enables evolution of microorganisms under conditions simulating actual environments (such as rhizosphere, gut, and fermenter conditions; Poltak and Cooper, 2011; Batstone et al., 2020). Through serial transfer under specific selective pressure, a part of microbial individuals can acquire advantageous mutations that enable them to adapt to given environments, eventually becoming dominant within the population (Walker et al., 2010; Cooper and Gales, 2018; Lenski, 2023). This method employs natural selection for driving microbial adaption to specific conditions, which is independence of genetic background or molecular regulation mechanisms, offering an appropriate strategy for breeding microbes applied in complex scene such as agricultural production.

Directed domestication have been widely applied in the fields of enzymatic and metabolic engineering; however, relevant research on rhizosphere microorganisms primarily focused on documenting adaptive phenotypic and genetic changes, rarely translating into practical application in agricultural production systems (Walker et al., 2010; Li et al., 2021; Tan et al., 2021; Hu et al., 2023). In the present study, we designed an *in situ* evolutionary experiment in the rhizosphere using the widely applied PGPR strain *Bacillus velezensis* SQR9, to identify evolved strains that exhibit superior growth-promoting effects compared to the ancestral strain. The developed strategy effectively enhances host compatibility and the functionality of existing plant-beneficial microbes, providing new insights for superior microbial fertilizer strains and optimizing agro-product effectiveness.

## 2 Materials and methods

### 2.1 Bacterial strains and culture conditions

The evolutionary experiment employed *Bacillus velezensis* SQR9, a well-characterized plant growth-promoting rhizobacterium from our laboratory collection. For reliable strain tracking and contamination control, we chromosomally integrated a GFP-chloramphenicol resistance plasmid prior to experimental procedures, according to the chemogenetic transformation method described by Chen et al. (2016). Initial cultures were prepared by streaking the bacterium onto LLB agar (10 g L^−1^ tryptone, 5 g L^−1^ yeast extract, 3 g L^−1^ NaCl, 20 g L^−1^ agar) containing 5 μg ml^−1^ chloramphenicol, followed by 30°C incubation for 16 h. Randomly selected colonies were propagated in chloramphenicol-supplemented LLB broth at 30°C with 200 rpm shaking for 12 h. Cells were pelleted, washed thrice with 10 mM MgSO_4_ to eliminate medium residues, and standardized to 10^5^ CFU ml^−1^ in sterile MgSO_4_ for evolutionary cultivation.

### 2.2 Host plant and growth conditions

Pepper (*Capsicum annuum* var. *conoides*) was used as the host plant. Seeds were soaked in deionized water for 12 h, then sterilized in 70% ethanol for 1 min and 6% sodium hypochlorite for 6 min. After sterilization, the seeds were rinsed three times with sterile water. Germination occurred on 0.25 × MS agar medium (pH 7.0) in square petri dishes under controlled conditions (22°C, 16/8-h photoperiod, 200 μmol·m^−2^·s^−1^ PAR) for 7 days. Seedlings were then transferred to sterile 750 ml vessels containing 100 g autoclaved vermiculite and 90 ml 0.25 × MS solution, with two plants per vessel to establish rhizosphere colonization system.

### 2.3 Design of the evolutionary experiment

Two seven-day-old sterile pepper seedlings were transplanted into sterilized vermiculite containers and inoculated with an initial bacterial suspension (10^5^ cells ml^−1^) in the rhizosphere, using a volume of 1 ml for the inoculum. After 1 week of cultivation, plant roots were transferred to centrifuge tubes containing 5 ml of 6 g L^−1^ NaCl solution with two sterilized 2-mm glass beads. Root-associated bacteria were dislodged by vortex mixing (1,500 rpm, 10 min). One milliliter of the resulting suspension was transferred to new seedling rhizosphere, and the residue underwent serial dilution for colony quantification. The transfer totally repeated for 20 cycles and maintained five independent replicate lineages throughout the experiment.

### 2.4 Root colonization assessment

After overnight activation on LLB agar, the single colonies of different evolved strains were inoculated into LLB broth supplemented with chloramphenicol. The cultures were incubated at 30°C on a shaking platform at 200 rpm for 12 h. The cells were washed three times with 10 mM sterile MgSO_4_ to remove residual culture medium. The final concentration was standardized to 10^5^ CFU/ml in sterile MgSO_4_.

Seven-day-old sterile pepper seedlings were transplanted into sterile vermiculite containers. One milliliter of the initial bacterial suspension was inoculated at the root zone. After 1 week of cultivation, plant roots were rinsed with sterile water. They were then transferred to 5 ml of 6 g/L NaCl solution and vortexed at 1,500 rpm for 10 min to recover root-attached bacteria. The resulting bacterial suspension was serially diluted and plated on LLB agar for colony counting.

### 2.5 Measurement of plant-associated traits by different evolved strains

At the end of the final cultivation cycle, nine colonies were randomly selected from each evolutionary lineage to evaluate the following functions:

*IAA production* Two μl overnight bacterial culture was inoculated into a 96-well plate containing 1 g L^−1^ tryptophan in the Landy medium, and incubate at 25°C, 100 rpm for 72 h. The culture was centrifuged at 3,000 rpm for 30 min, and the cell-free supernatant was obtained by filtering through a 0.22 μm membrane. The supernatant was reacted with R1 reagent (FeCl₃ 312 g L^−1^, H₂SO₄ 7.9 M) in the dark for 30 min, and the absorbance was measured at 530 nm (n = 6).

*Siderophore production* Two μl overnight culture was inoculated into a 96-well plate containing 190 μl of iron-limited MKB liquid medium. The plate was incubated at 30°C with shaking at 170 rpm for 48 h. After incubation, the cultures were centrifuged at 3,000 rpm for 30 min, and the supernatant was filtered through a 0.22 μm membrane. The sterile supernatant was mixed with an equal volume of CAS detection reagent and incubated for 2 h. The absorbance (A) of the samples was then measured at 630 nm, while the A_630_ (Ar) of the control group was prepared following the same method with un-inoculated medium. The iron carrier units (SU) were calculated using the given formula: SU = 1 − (A/Ar; Schwyn and Neilands, 1987).

*Biofilm Formation* Two μl overnight culture was inoculated into a 96-well plate containing 198 μl of MSgg medium and incubated at 30°C for 48 h. When sampling, the medium beneath the biofilm was carefully aspirated using a micropipette, and the biofilm was then washed twice with 150 μl of phosphate buffer. Next, 0.1% (w/v) crystal violet solution was added and allowed to stain for 30 min. After discarding the staining solution, the biofilm was rinsed twice with deionized water. Finally, 150 μl of an ethanol/acetone solution (80:20, v/v) was added to dissolve the dye attached to the biofilm, and the absorbance was measured at 570 nm to quantify the biofilm formation.

### 2.6 Evaluation of the pepper growth-promotion effects by evolved bacteria

The ancestral strain or evolved strains were inoculated into LLB liquid medium. After shaking at 30°C and 170 rpm for 24 h, the cells were collected by centrifugation at 6,000 rpm for 10 min. The cells were then resuspended in 0.25 × MS culture medium to adjust the concentration to about 5.0 × 10^8^ CFU ml^−1^ for inoculation.

*Hydroponic Experiment* Roots of the 7-day-old pepper seedlings (cultivated as described above) were gently rinsed with sterile water to remove residual agar medium, then the seedlings were transferred to 50 ml Erlenmeyer flasks containing 24 ml 0.25 × MS solution pre-inoculated with 1 ml bacterial suspension (ancestral or evolved strains). Flasks were maintained in greenhouse under 25°C with 16/8-h photoperiod for 7 days. Plant growth parameters including shoot fresh weight, root fresh weight, and lateral root count were subsequently quantified.

*Pot experiment* 25-day-old pepper seedlings were transplanted into sterilized pots. Each pot contained one plant and 3 kg of unsterilized natural soil. Bacterial suspensions with a final concentration of ≥1.0 × 10^9^ CFU were evenly applied around the seedling roots, using a volume of 10 ml for the inoculum. The soil had the following properties: pH 7.83, organic matter content of 12.08 g/kg, total nitrogen of 71.12 mg/kg, total phosphorus of 0.85 g/kg, total potassium of 16.83 g/kg, available phosphorus of 55.55 mg/kg, and available potassium of 137.33 mg/kg. Plants were maintained at 25°C with a 16/8-h light/dark cycle. The experiment included eight treatments as sterile water irrigation (CK), ancestral strain inoculation (WT), and inoculations with different evolved strains (9P15, 9P16, 9P19, 9P27, 9P38, 9P41); and six biological replicates were included in each treatment. After 25 days of cultivation, samples were collected to measure plant height, root length, shoot fresh weight, root fresh weight, and chlorophyll SPAD values.

### 2.7 Re-sequence analysis of evolved strain

The evolved strain 9P41 that showed the best plant growth-promotion performance, was selected for genome resequencing. The frozen bacterial solution was taken out from the refrigerator and spread on LLB plate containing antibiotics. After extracting the genomic DNA of the evolutionary strain and the ancestral strain, resequencing was performed following the processes as: (1) DNA quality assessment; (2) fragmentation of DNA using ultrasonic methods to achieve sizes between 300–500 bp; (3) end repair of DNA fragments using T4 DNA polymerase, adding an A base at the 3′ end to create sticky ends; (4) ligation of DNA adapters containing index sequences to the sticky ends, following the base pairing principle, for Illumina sequencing; (5) selection of specific length fragment sequences using magnetic beads; (6) PCR amplification of the target fragments, adding index sequences at both ends for downstream library preparation and sequencing; (7) fixation of the sequencing library onto a sequencing chip via bridge PCR; (8) paired-end sequencing on the Illumina NovaSeq platform, executed as 2 × 150 bp. The mutation screening criteria are as follows: the total coverage of mutation site detection is at least 2 times (i.e., covered by at least 2 reads). The minimum frequency of the gene mutation in the population is ≥5%. The gene locus mutation is a non-synonymous mutation.

### 2.8 Data analysis

Data analysis and visualization were performed using R (v4.1.3) and GraphPad Prism 9. Statistical significance was set at *p* < 0.05. Multiple group comparisons employed two-way ANOVA with Tukey’s *post hoc* tests for pairwise comparisons.

## 3 Results

### 3.1 *In situ* transfer in rhizosphere enhances host affinity of PGPR agent *B. velezensis* SQR9

**Figure 1:**
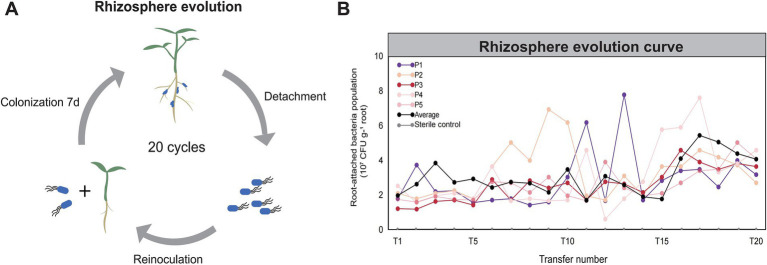
Directed evolution of PGPR *Bacillus velezensis* SQR9 in pepper rhizosphere. **(A)** Schematic diagram of the directed evolution of strain SQR9 in pepper rhizosphere. **(B)** Evolutionary dynamics of *B. velezensis* SQR9. The horizontal coordinate is the number of transfers, and the vertical coordinate is the colonization amount of the bacteria in rhizosphere. Five parallel evolutionary lineages were designed in the experiment. “Average” represents the mean value of the colonization amounts across all lineages.

In order to improve the rhizosphere adaptation and probiotic function of a model PGPR strain *B. velezensis* SQR9, we designed an *in situ* rhizosphere experimental evolution by continuous domestication of the bacteria in pepper rhizosphere (see Materials and Methods, Figure 1A), resulting in approximately 160 generations of cumulative evolution. Five parallel evolutionary lineages (designated as P1-P5) were established, and the bacterial root colonization capacity was quantified after each transfer via dilution plating. The ancestral strain initially colonized plant roots at approximately 1.7 × 10^7^ CFU g^−1^ root (Figure 1B), and the average colonization level significantly evolved during the transfer process: colonization slowly increased in the early stage before the fifth transfer (pre-T5), experienced fluctuations during the mid-stage (T10 to T14), and had significant growth in the later stage (T14 to T17). By T18 and T20, bacterial colonization stabilized across all lineages, ranging from 2.6 × 10^7^ to 5.0 × 10^7^ CFU g^−1^ root. This change marked an improvement of 1.5 to 2.9 times compared to the ancestral strain.

### 3.2 Preliminary screening of evolved strains with improved plant-associated traits

**Figure 2:**
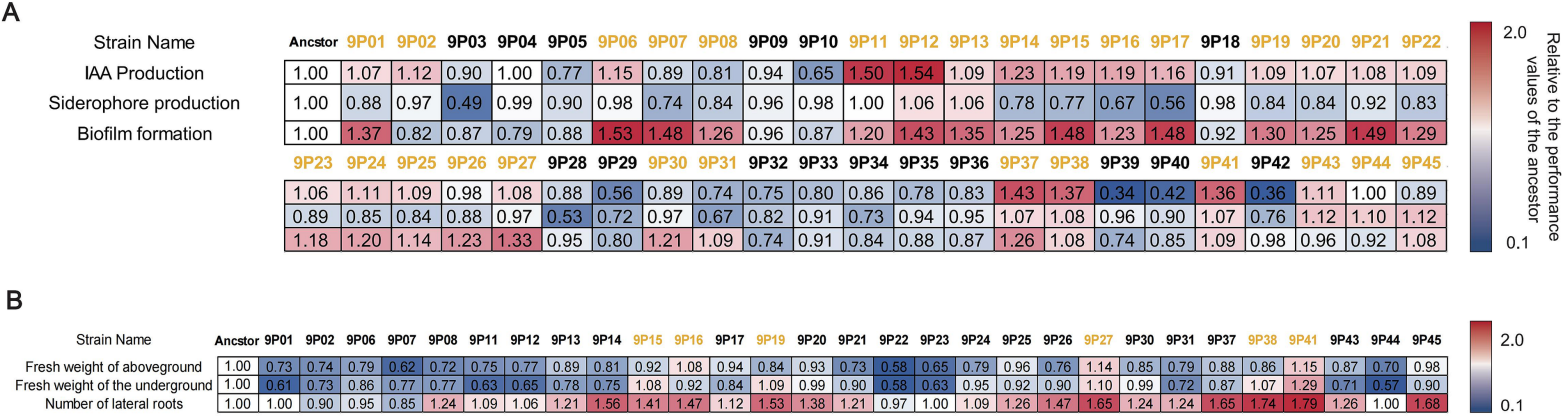
Multi-stage screening of rhizosphere evolved strains of pepper according to plant-associated traits and growth-promoting performance. **(A)** Screening of plant-associated traits of bacteria evolved in pepper rhizosphere. The horizontal labels represent ancestral strains and evolved strains (9 strains were selected from each lineage, totaling 45 strains, labeled as 9Pn). The vertical labels represent plant-associated traits, including IAA production, siderophore production, and biofilm formation. **(B)** Screening of rhizosphere-evolving strains based on plant growth promotion effects in hydroponic system. Horizontal labels indicate ancestral and evolved strains (labeled as 9Pn), while vertical labels represent plant growth promotion. The values in the heat map represent the relative ratio of the given index between the evolved strain and the ancestor; the yellow means that an evolved strain exhibits at least one superior phenotype over the ancestral strain. This phenotype shows an improvement with an increase of ≥5%. These difference is statistically significant (*p* < 0.05).

Based on the five evolved *B. velezensis* SQR9 lineages cultured on LLB agar, nine colonies were randomly selected from each lineage (totaling 45 evolved strains, labeled as 9P1 ~ 9P45). These strains were assessed for three plant-associated traits as indole-3-acetic acid (IAA) production, siderophore production, and biofilm formation (Figure 2A). The ancestral strain SQR9 displayed baseline values of IAA secretion by 12.03 mg L^−1^, siderophore production of 0.399 units (SU), and biofilm formation ability of OD_570_ as 1.779. Among the picked 45 evolved strains, 22 exhibited enhanced IAA production than the ancestor (6.1% ~ 54.1% increase, maximum production of 18.54 mg L^−1^ by 9P12), nine showed improved siderophore secretion (6.2% ~ 12.3% increase, peak SU of 0.448 by 9P45), and 26 demonstrated superior biofilm formation (8.2% ~ 53.5% increase, highest OD_570_ as 2.731 by 9P6). As a result, 29 strains showing improvements in at least one trait (with an increase of ≥5%) were selected for further evaluating the plant growth promotion effects in hydroponic experiment.

### 3.3 Secondary screening of evolved strains based on plant growth-promotion efficacy in hydroponic system

The selected 29 evolved strains were further evaluated for their plant growth-promotion efficacy using a hydroponic system. Pepper plants inoculated with ancestral strain SQR9 revealed baseline performance with an aboveground biomass of 0.175 g, a root biomass of 0.079 g, and an average of 34 lateral roots per plant. Among the evolved strains, 3 demonstrated improved the plant aboveground biomass compared to the ancestral strain (8.0% ~ 14.9% increase, maximum 0.201 g by 9P41); five strains announced enhanced root biomass (8.3% ~ 29.1% increase, maximum 0.102 g by 9P41), and 24 strains exhibited an increase in lateral root formation (5.8% ~ 79.4% increase, maximum 61 roots per plant by 9P41; Figure 2B). As a result, six evolved strains (9P15, 9P16, 9P19, 9P27, 9P38, and 9P41) each showing an advantage in at least one plant growth parameter than the ancestor (with an increase of ≥5%), were selected for assessing their plant growth-promoting capacity in the following pot experiment.

### 3.4 Pot experiment elected strain 9P41 as a significant evolved plant growth-promoter

**Figure 3:**
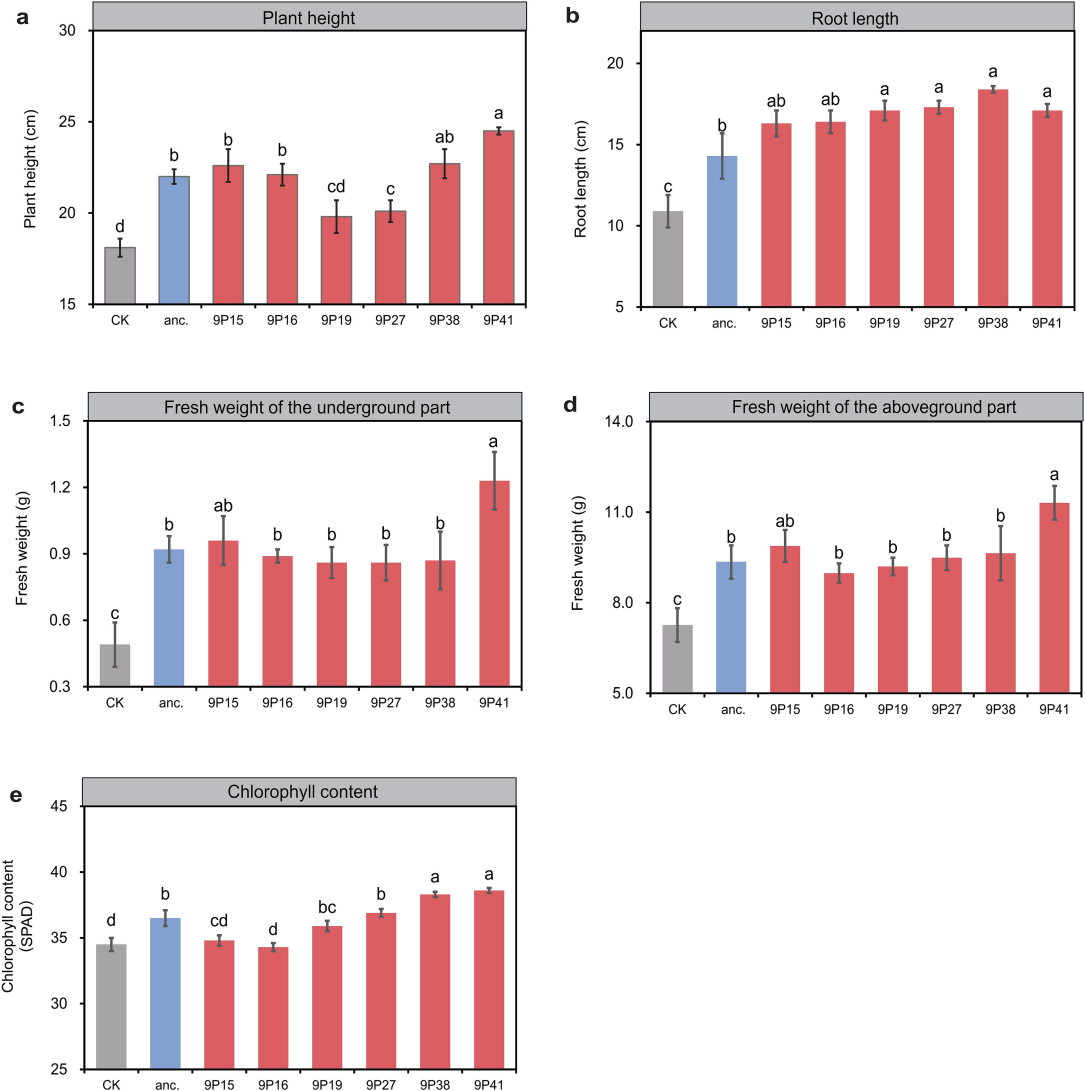
The evolved strains exhibit a stronger growth-promoting effect in pot experiment. **(A)** The plant height of potted peppers. **(B)** The root length of potted peppers. **(C)** The fresh weight of the underground parts of potted peppers. **(D)** The fresh weight of the above-ground parts of potted peppers. **(E)** The chlorophyll content of potted peppers. The horizontal axis represents different treatments, including no inoculation (CK), inoculation with ancestral strain (anc.), and inoculation with evolved strain (9Pn). Different letters indicate significant differences based on the one-way ANOVA and Tukey’s test. n = 6. *p* < 0.05.

To better simulate the field production condition, finally we inoculated the 6 selected evolved strains into 25-day-old pepper plants in pots for growth promotion assessment. After 25 days, all treatments with evolved strains outperformed the ancestral strain in at least one trait (Figure 3). Specifically, evolved strain 9P41 pronounced the best plant growth-promotion performance, with an 11.4% increase in plant height than the ancestor-inoculated plants (24.5 vs. 22.0 cm), a 28.7% increase in root length (18.4 vs. 14.3 cm), a 21.0% increase in aboveground biomass (11.31 vs. 9.35 g), a 29.1% increase of underground biomass (1.23 vs. 0.92 g), and a 5.8% increase in chlorophyll SPAD value (38.6 vs. 36.5). Other evolved strains also displayed specific improvements, such as 9P38-inoculated plants showed an increase of 28.7% in root length than the ancestor, while 9P15 demonstrated a 4.3% increase in underground biomass. These results indicate that rhizosphere domestication selects strains with evolved plant-associated functions, with 9P41 emerging as the most effective plant growth promoter.

### 3.5 Genome resequencing analysis revealed gene mutations may affect enhanced traits

Given the outstanding plant growth-promotion performance by the evolved strain 9P41, we aimed to identify the genetic determinants of its enhanced traits through whole-genome resequencing. Comparative genomic analysis with its ancestor revealed three mutations in coding regions as *mlnD*, *smc*, and *fhuC* (Table 1). In detail, the *mlnD* gene encodes a type I polyketide synthase that catalyzes the biosynthesis of polyketides through sequential condensation of carboxylic acid precursors (Liang et al., 2019). In the *Bacillus* genus, these polyketide derivatives, such as macrolides, exhibit broad-spectrum antibacterial activity against both Gram-positive and Gram-negative pathogens by interfering with cell division and cell wall synthesis (Jun et al., 2012). The product of the *smc* gene forms a conserved ATP-dependent protein complex essential for chromosome organization, mediating ATP-driven DNA loop extrusion to maintain genomic integrity during replication (Jun et al., 2012). The *fhuC* gene encodes a key ATPase subunit of an ABC transporter that is crucial for the uptake of iron–siderophore complexes, where ATP hydrolysis provides the energy necessary for transmembrane transport (Athanasios and Wolfgang, 1998; Wenxin and Hongjin, 2021). Collectively, three genes (*mlnD*, *smc*, and *fhuC*) with potential links to plant-microbe interactions, are identified to be mutated during the evolution process by strain 9P41.

**Table 1:** Mutated genes of the evolutionary strain 9P41.

Mutant gene	Mutation location	Description
*mlnD*	G70A: E24K	type I polyketide synthase
*smc*	C1715T: A572V	chromosome segregation protein SMC
*fhuC*	G730T: V244L	ferrichrome ABC transporter

Genome resequencing and comparative analysis with the ancestral strain identified three mutations of 9P41. The “Mutant gene” column is the name of the mutated gene; the “Mutation location” column is the specific mutation site, including the changed base and amino acid; the “Description” column is the functional description of the mutated gene.

## 4 Discussion

The present domestication framework provides an alternative strategy for enhancing field performance of laboratory-selected PGPR strains. Through rhizosphere adaptation and a multi-stage screening of plant-associated parameters, we successfully identified a couple of bacterial variants with improved root colonization ability and multi-trait enhancements, including beneficial functions such as plant hormone synthesis and biofilm formation. Importantly, strain 9P41 demonstrates significant improvements in ecological adaptability and functional stability—two critical factors often compromised in traditional microbial fertilizer applications. This evolution-driven strategy extends the application of adaptive microbial domestication beyond industrial enzyme optimization, effectively relieving the issue of “laboratory-field efficacy disconnect” that affects the performance of agricultural microbial inoculant (Li et al., 2021; Tan et al., 2021).

Under specific host selection pressure during continuous *in situ* transfer, the colonization of *B. velezensis* SQR9 among five evolved lineages became stable in the later evolutionary stages (T18 ~ T20), indicating that the domestication period was sufficient and validate the feasibility of the overall framework (Lenski, 2017). It seems that the increase in bacterial colonization (from 1.7 × 10^7^ CFU g-1 root to 5.0 × 10^7^ CFU g^−1^ root; 2.9-fold) still has considerable potential. Evolution experiments in other studies similarly involved inoculating strains into the rhizosphere of healthy plants without additional stress, and the final improvement in root adaptability was comparative with our study (Li et al., 2021; Hu et al., 2023). In contrast, evolution experiments designed under strong/specific selective pressure bred microbes with significant enhanced adaptation. The increase in microbial population abundance allows for greater resource acquisition in the rhizosphere and helps maintain dominance within the microbial community (Helen et al., 2010; Guttman et al., 2012; Alice et al., 2014). Future domestication design can improve by introducing additional selective stresses such as pathogen infection or salt stress, compelling the target strains to be more intimate with plants, especially with enhanced beneficial functions that their host expect (Guttman et al., 2012; Nan et al., 2023; Megan et al., 2024).

We propose that the enhanced root colonization of evolved strains may result from the enhancement of plant-associated functions by microorganisms. Therefore, we select three plant-associated traits for preliminary screening of the evolved communities, as synthesis of IAA (enhanced in 48.9% of evolved strains) that regulates root growth, production of siderophores (20.0%) that enhance iron absorption in deficient soils, and biofilm formation (57.8%) that facilitate bacterial colonization (Xu et al., 2019; Hassan and Bernard, 2024; Nanqi et al., 2024). Interestingly, bacterial colonization triggers immune responses and ROS, which enhance bacterial IAA production. IAA improves bacterial survival and colonization, enabling plant health promotion (Wein, et al., 2019; Tzipilevich et al., 2019). It should be noticed that that only a portion of evolved strains with improved traits consistently promoted plant growth in hydroponic and pot experiments. This indicates that many other factors may influence the growth-promoting performance of the strains, possibly including synergistic improvements in multifunctionality or other traits that we have not detected (Megan et al., 2024). Specifically, strain 9P41, which enhances all three plant-related traits, performed better than other evolved strains, while strains with only a single trait improvement, such as 9P45 with siderophore advantage, may lack the multifunctionality required to thrive in the rhizosphere. In conclusion, an increase in population abundance or an enhancement of plant-related functions can improve the overall growth-promoting performance of the evolved populations.

Whole-genome resequencing revealed nonsynonymous mutations in *mlnD*, *smc*, and *fhuC* linked to rhizosphere adaptation of the evolved strain 9P41. The *mlnD* mutation, located in the polyketide synthesis domain, requires further validation to determine if it enhances or reduces antibiotic production; Because no other strains were added in our evolution experiment, it is deduced that this mutation may save costs in antibiotic production, allowing more resources to enhance other physiological activities such as biofilm formation (Jun et al., 2012). The *smc* mutation may influence chromosome organization and plays an important role in facilitating DNA replication and maintaining genomic stability (Anna et al., 2021; Vázquez et al., 2021). The *fhuC* mutation was reported to be correlated with increased yield of siderophore, therefore releasing more available iron into the soil (Teresa et al., 2000; Wenxin and Hongjin, 2021), not only directly supports bacterial growth and adaptation, but also regulates root development to enhance exudate production and thereby boost their colonization (Nanqi et al., 2024). Further validation is needed for confirming the detailed roles of these mutations in the enhanced bacterial phenotypes, providing potential target sites for genetic modification.

## 5 Conclusion

In summary, this study proposes a rhizosphere domestication strategy to breed PGPR strain with improved rhizosphere colonization and plant growth-promotion ability, which can serve the development of microbial fertilizers and relevant industry. Future research can develop novel bio-organic fertilizers with the evolved 9P41, and also upgrade the domestication route for improving breeding efficiency and accuracy. We also propose this framework has potential for developing functional microbial communities through targeted environmental adaptation.

## Data Availability

The original contributions presented in the study are publicly available. This data can be found here: https://www.ncbi.nlm.nih.gov/, accession number PRJNA1293123.
